# EUS‐Guided Gastroenterostomy for Malignant Gastric Outlet Obstruction: Morphological Classification to Prevent Stent Misdeployment

**DOI:** 10.1111/den.70235

**Published:** 2026-07-17

**Authors:** Yu‐Ting Kuo, Chi‐Ying Yang, Jiann‐Hwa Chen, Szu‐Chia Liao, Cheng‐Lin Hsieh, Chia‐Hsien Wu, Meng‐Ying Lin, Chen‐Shuan Chung, Kuan‐Chih Chen, Mu‐Hsien Lee, Hsiang‐Yao Shih, Jung‐Chun Lin, Cheuk‐Kay Sun, Ming‐Chang Tsai, Hsiu‐Po Wang, the Taiwan EUS‐GE Study Group (TEGESG)

**Affiliations:** ^1^ Division of Endoscopy, Department of Integrated Diagnostics & Therapeutics National Taiwan University Hospital, National Taiwan University Taipei Taiwan; ^2^ Department of Internal Medicine, College of Medicine National Taiwan University Taipei Taiwan; ^3^ Department of Internal Medicine, Center for Digestive Medicine China Medical University Hospital, China Medical University Taichung Taiwan; ^4^ Division of Gastroenterology and Hepatology, Department of Internal Medicine Taipei Tzu Chi Hospital & School of Medicine, Tzu Chi University Hualien Taiwan; ^5^ Division of Gastroenterology and Hepatology, Department of Internal Medicine Taichung Veterans General Hospital Taichung Taiwan; ^6^ Department of Post‐Baccalaureate Medicine, College of Medicine National Chung Hsing University Taichung Taiwan; ^7^ Division of Gastroenterology and Hepatology, Department of Internal Medicine National Taiwan University Hospital Hsin‐Chu Branch Hsinchu County Taiwan; ^8^ Division of Gastroenterology and Hepatology, Department of Internal Medicine Taitung Mackay Memorial Hospital Taitung Taiwan; ^9^ Department of Internal Medicine, College of Medicine National Cheng Kung University Hospital, National Cheng Kung University Tainan Taiwan; ^10^ Division of Gastroenterology and Hepatology, Department of Internal Medicine Far Eastern Memorial Hospital New Taipei City Taiwan; ^11^ Department of Electrical Engineering Yuan Ze University Taoyuan Taiwan; ^12^ Graduate Institute of Biomedical Electronics and Bioinformatics National Taiwan University Taipei Taiwan; ^13^ Department of Gastroenterology and Hepatology Linkou Medical Center, Chang Gung Memorial Hospital and, Chang Gung University College of Medicine Taoyuan Taiwan; ^14^ Division of Gastroenterology, Department of Internal Medicine Kaohsiung Medical University Hospital Kaohsiung Taiwan; ^15^ Division of Gastroenterology, Department of Internal Medicine Tri‐Service General Hospital, National Defense Medical University Taipei Taiwan; ^16^ Division of Hepatology and Gastroenterology, Department of Internal Medicine Shin Kong Wu Ho‐Su Memorial Hospital Taipei Taiwan; ^17^ Division of Gastroenterology and Hepatology, Department of Internal Medicine Chung Shan Medical University Hospital Taichung Taiwan; ^18^ School of Medicine Chung Shan Medical University Hospital Taichung Taiwan; ^19^ Division of Gastroenterology and Hepatology, Department of Internal Medicine National Taiwan University Hospital Taipei Taiwan; ^20^ Division of Gastroenterology, Center of Excellence for Innovation and Endoscopy in Gastrointestinal Oncology, Department of Medicine, Faculty of Medicine Chulalongkorn University Bangkok Thailand

## Abstract

**Objective:**

Stent misdeployment is the most critical adverse event (AE) during endoscopic ultrasound‐guided gastroenterostomy (EUS‐GE). This study aimed to identify predictors of misdeployment in EUS‐GE for malignant gastric outlet obstruction (mGOO).

**Methods:**

This multicenter observational study utilized a prospectively maintained registry of consecutive patients undergoing EUS‐GE for mGOO at 13 tertiary centers in Taiwan, with mixed retrospective‐prospective assessment of fluoroscopic morphology (FM) of the gastrointestinal tract near the ligament of Treitz (four types) and EUS morphology (EM) of the target bowel (three types). The primary outcome was the misdeployment. Multivariable analysis identified predictors of misdeployment and survival.

**Results:**

Between July 2020 and June 2025, 165 patients were enrolled. Technical and clinical success rates were 98.2% and 95.2%, respectively. Overall AE rate and misdeployment rates were 7.3% and 4.8%, respectively. Same‐session endoscopic salvage succeeded in 87.5% of misdeployment cases. EM type C (cross‐sectional bowel view) carried the highest misdeployment risk (33%) and was the sole independent predictor (adjusted odds ratio 16.9; *p* = 0.001). FM provided essential complementary risk stratification; FM type II was more common among misdeployment cases (25.0% vs. 3.8%; *p* = 0.030). Implementation of a stepwise strategy in 2023—prioritizing EM type A/B, avoiding type C, and minimizing stomach–bowel distance—resulted in zero misdeployment. Independent predictors of poor survival included pancreatic cancer, malnutrition, poor performance status, ascites, metastasis, and misdeployment.

**Conclusion:**

Stent misdeployment is a significant complication adversely impacting survival. Utilizing integrated EM and FM risk stratification within a stepwise strategy can minimize misdeployment and optimize clinical outcomes.

**Trial Registration:**

ClinicalTrials.gov: NCT07230665

## 1 Introduction

Endoscopic ultrasound‐guided gastroenterostomy (EUS‐GE) has emerged as a minimally invasive alternative to surgical gastroenterostomy (SGE) and endoscopic enteral stenting (ES) for gastric outlet obstruction (GOO) [1]. By utilizing a lumen‐apposing metal stent (LAMS) to bypass the obstruction, EUS‐GE combines surgical durability with the minimally invasive nature of endoscopy. Recent evidence, including a randomized controlled trial (RCT) and a network meta‐analysis, suggests EUS‐GE offers superior stent patency and fewer reinterventions than ES, with a safety profile surpassing both traditional modalities in malignant GOO (mGOO) [2, 3].

Despite these advantages, EUS‐GE remains technically demanding. Approximately 15% of cases involve adverse events (AEs), primarily stent misdeployment [4]. Unlike other interventional EUS procedures targeting fixed anatomical structures, EUS‐GE involves a mobile jejunal loop, increasing the inherent risk of misdeployment—ranging from 4.6% to 10%—even among experienced endoscopists [4, 5]. To date, predictors of misdeployment remain poorly investigated, and a standardized morphological classification is lacking. To address this gap, we proposed a novel classification system integrating fluoroscopic and EUS imaging features to stratify misdeployment risk and evaluated its utility in a prospective multicenter cohort.

## 2 Methods

### 2.1 Study Design

This multicenter observational study was conducted using a prospectively maintained registry of consecutive adults (≥ 18 years) undergoing EUS‐GE for unresectable mGOO across 13 academic centers in Taiwan between July 2020 and June 2025. The registry was originally established to evaluate clinical outcomes following EUS‐GE. After development and refinement of the FM and EM classifications, these variables were prospectively recorded in subsequent patients, whereas earlier cases were retrospectively classified using archived fluoroscopic and EUS images reviewed by the investigators. Accordingly, the present study represents a mixed retrospective–prospective assessment performed within a prospectively maintained multicenter registry. Patients were excluded if they had multilevel obstruction, linitis plastica, life expectancy < 1 month, uncorrectable coagulopathy, pregnancy, or follow‐up < 30 days. All participating endoscopists had performed ≥ 10 LAMS‐based interventional EUS procedures. To ensure procedural consistency, the Taiwan EUS‐GE Study Group (TEGESG) implemented standardized peri‐procedural protocols (Table S1). To mitigate the learning curve, the first five cases at each center were supervised on‐site or via tele‐mentoring by experts from the lead institution (National Taiwan University Hospital; KYT or WHP). Monthly webinars were held to review enrollments and manage technical challenges. Institutional Review Board approval was obtained at all sites (NCT07230665).

### 2.2 Endoscopic Ultrasound‐Guided Gastroenterostomy

EUS‐GE followed the wireless EUS‐GE simplified technique (WEST) [6] under general anesthesia with endotracheal intubation and prophylactic antibiotics. After guidewire passage across the obstruction, contrast was injected to delineate fluoroscopic anatomy near the ligament of Treitz. A nasobiliary tube was then placed to distend the target jejunum with saline, contrast, and indigo carmine. Following glucagon administration to minimize peristalsis, an electrocautery‐enhanced LAMS (Hot AXIOS, 20 mm diameter, 10 mm length; Boston Scientific, Marlborough, MA, USA) was deployed under combined EUS and fluoroscopic guidance.

### 2.3 Fluoroscopic Morphological Classification (FM)

**FIGURE 1:**
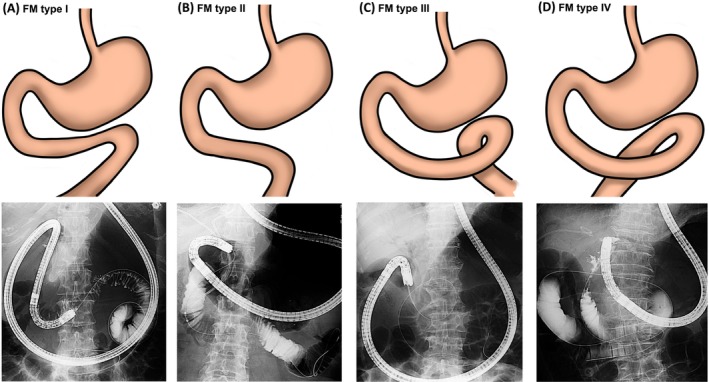
Fluoroscopic morphological classification (FM) of the gastrointestinal tract around the ligament of Treitz. (A) Type I, the fourth portion of the duodenum approaches the stomach and joins the jejunum without forming a loop. (B) Type II, the fourth portion of the duodenum does not approach the stomach and joins the jejunum without forming a loop. (C) Type III, the fourth portion of the duodenum forms a loop to the jejunum on the left side of the abdomen. (D) Type IV, the fourth portion of the duodenum forms a loop to the jejunum on the right side of the abdomen.

With the patient supine, FM near the ligament of Treitz was classified into four types (Figure 1): Type I, the fourth portion of the duodenum (D_4_) approached the stomach and joined the jejunum without forming a loop; Type II, D_4_ did not approach the stomach and joined the jejunum without loop formation; Type III, D_4_ formed a loop toward the left abdomen; and Type IV, D_4_ formed a loop toward the right abdomen.

### 2.4 EUS Morphological Classification (EM)

**FIGURE 2:**
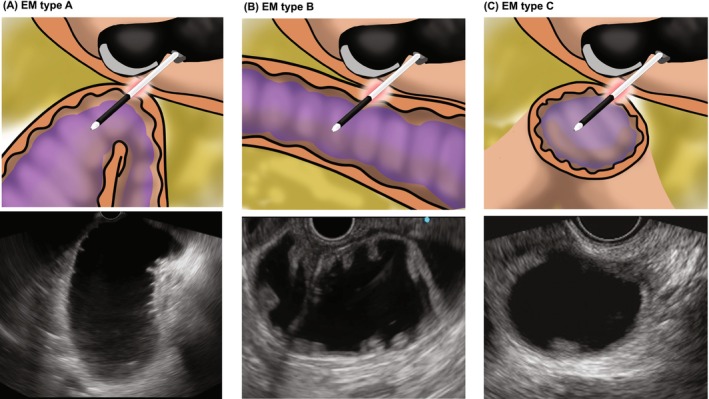
Endoscopic ultrasound (EUS)–based morphological classification (EM) of the targeted bowel loop. (A) Type A, the orientation of the target bowel lumen is along the longitudinal axis, which is perpendicular to the EUS probe. (B) Type B, the orientation of the target bowel lumen is along the horizontal axis, which is parallel to the EUS probe. (C) Type C, the target bowel lumen is seen in cross‐section as a circle.

EM of the targeted bowel was classified into three types (Figure 2): Type A, the bowel was visualized longitudinally and perpendicular to the ultrasound beam; Type B, longitudinally and parallel to the ultrasound probe; and Type C, in cross‐section as a circular structure (Figure 2C).

### 2.5 Outcome Assessments and Definition

**FIGURE 3:**
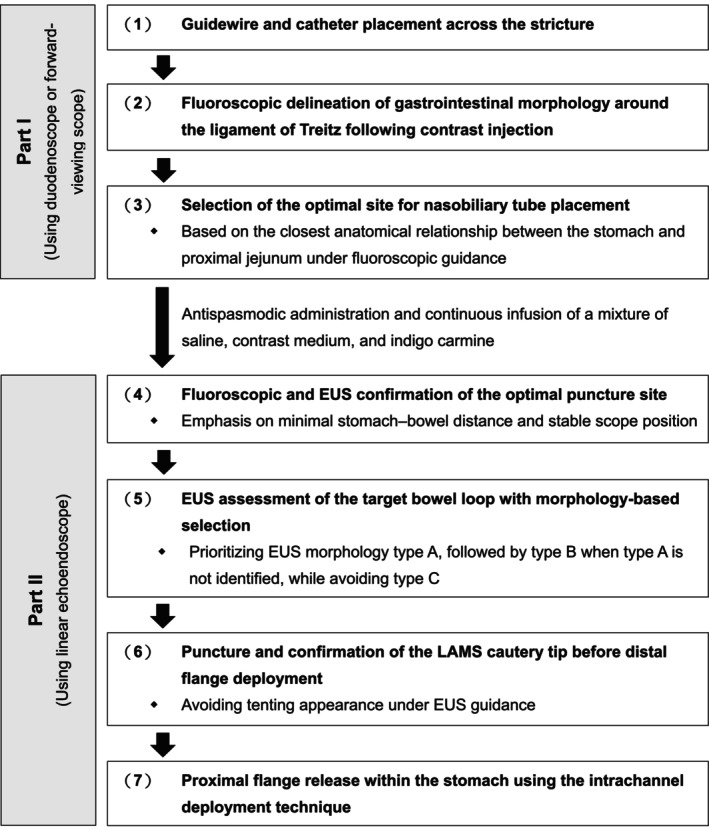
Stepwise strategy for puncture‐site determination, target bowel selection, and deployment of a lumen‐apposing metal stent (LAMS) during endoscopic ultrasound–guided gastroenterostomy.

Clinical, procedural, and outcome data were systematically collected throughout the study period. Interobserver agreement for FM and EM was assessed using the *k*‐coefficient based on independent interpretations by three blinded reviewers (KYT, YCY, and HCL). The primary outcome was intraprocedural stent misdeployment, classified into four types: Type I, distal flange in the peritoneum without bowel penetration; Type II, distal flange in the peritoneum following bowel penetration and migration; Type III, distal flange in the small bowel, but proximal flange in the peritoneum; and Type IV, distal flange in the colon, creating a gastrocolic anastomosis [5]. Secondary outcomes included technical success (successful stent placement bypassing obstruction), clinical success (≥ 2 point improvement in the GOO score (GOOS) after procedure), reintervention rate (the need for additional endoscopic treatment due to recurrent GOO symptoms), overall AEs, and survival. GOOS categorized oral intake into four levels: 0 (fasting), 1 (liquid diet), 2 (soft diet), and 3 (normal/low‐residue diet) [7]. AEs were classified and graded according to the American Society for Gastrointestinal Endoscopy (ASGE) lexicon [8]. Following implementation of the stepwise strategy in 2023—prioritizing EM type A/B, avoiding type C, and minimizing stomach–bowel distance—outcomes were compared between the 2020–2022 and 2023–2025 cohorts (Figure 3).

### 2.6 Statistical Analysis

Categorical variables were compared using the Fisher's exact test, while continuous variables were analyzed using Student's *t*‐test or Mann–Whitney *U* test, as appropriate. Interobserver agreement for FM and EM was quantified using the *κ*‐coefficient, with values > 0.80 representing excellent agreement. Interval from procedure to reintervention and survival were estimated via Kaplan–Meier analysis, with censoring at death or last follow‐up. Logistic regression identified predictors of misdeployment; Cox proportional hazards models evaluated factors associated with survival. Results are reported as odds ratios (OR) or hazard ratios (HR) with 95% confidence intervals (CI). Variables with a *p* value of < 0.20 in univariable analysis were included in the multivariable model. Predefined clinical categories were established as follows: endoscopist proficiency was dichotomized into proficient and nonproficient cohorts at a threshold of ≥ 25 EUS‐GE procedures [9]. Preprocedural body mass index (BMI, kg/m^2^) was stratified into underweight (< 19), normal (19–24.9), overweight (25–29.9), and obese (≥ 30). Nutritional status was categorized by serum albumin (g/dL): normal (≥ 3.5), mild (3.0–3.4), moderate (2.5–2.9), and severe malnutrition (< 2.5). Performance status was evaluated via the Eastern Cooperative Oncology Group (ECOG) scale, dichotomized as 0–1 (no or mild limitation) versus 2–4 (moderate to severe limitation) [10]. Statistical analyses were performed using Stata version 15 (StataCorp, College station, TX, USA). All tests were two‐tailed, with *p* < 0.05 considered statistically significant.

## 3 Results

### 3.1 Patient Population and Procedure Details (Table 1)

A total of 165 consecutive patients undergoing EUS‐GE for mGOO were enrolled. The mean age was 66.9 years, and 49.7% were male. Pancreatic cancer was the predominant etiology (66.1%). Technical and clinical success rates were 98.2% and 95.2%, respectively, with a median procedure time of 39 min (interquartile range [IQR], 29–56) and a median saline injection volume of 700 mL (IQR, 450–1050).

**TABLE 1:** Baseline characteristics of patients undergoing endoscopic ultrasound‐guided gastroenterostomy (EUS‐GE) and procedure details.

	All patients (*n* = 165)
Baseline characteristics	
Male, *n* (%)	82 (49.7)
Age, years, mean ± SD	66.9 ± 14.1
BMI, median (IQR)	19.9 (17.7–22.5)
Albumin, g/dL, mean ± SD	3.3 ± 0.6
ECOG, *n* (%)	
None/mild	69 (41.8)
Moderate/severe	96 (58.2)
Cancer type, *n* (%)	
Nonpancreatic cancer	56 (33.9)
Pancreatic cancer	109 (66.1)
Presence of distal metastasis, *n* (%)	97 (58.8)
Presence of ascites, *n* (%)	73 (44.2)
Presence of peritoneal seeding, *n* (%)	52 (31.5)
Procedure details and outcomes	
Technical success, *n* (%)	162 (98.2)
Clinical success, *n* (%)	157 (95.2)
Procedure time, min, median (IQR)	39 (29–56)
Saline injection amount, mL, median (IQR)	700 (450–1050)
Fluoroscopic morphology (FM), *n* (%)	
Type I	91 (55.2)
Type II	8 (4.8)
Type III	45 (27.3)
Type IV	21 (12.7)
EUS morphology (EM), *n* (%)	
Type A	79 (47.9)
Type B	71 (43)
Type C	15 (9.1)
Follow‐up duration, days, median (IQR)	96 (53–211)
Interval from procedure to reintervention, days, median (IQR)	767 (389‐NR)^a^
Reintervention, *n* (%)	21 (12.7)
Adverse event (AE), *n* (%)	12 (7.3)
Stent misdeployment	8 (4.8)
Type I misdeployment	4 (2.4)
Type II misdeployment	4 (2.4)
Bleeding	2 (1.2)
Migration	2 (1.2)
ASGE severity grading of AE, *n* (%)	
Mild	9 (75)
Moderate	3 (25)
Survival length, days, median (IQR)	114 (58–226)

Abbreviations: AE, adverse event; BMI, body mass index; ECOG, eastern cooperative oncology group; EUS, endoscopic ultrasound; IQR, interquartile range; NR, not reached; SD, standard deviation.

^a^ Kaplan–Meier analysis.

Misdeployment occurred in eight patients (4.8%; four type I, four type II). All were managed via endoscopic closure using over‐the‐scope clips. According to the ASGE lexicon, most misdeployments were mild (*n* = 5, 62.5%) or moderate (*n* = 3, 37.5%). Postmisdeployments complications included peritonitis (*n* = 2) requiring prolonged antibiotics and aspiration pneumonia (*n* = 1). Same‐session salvage for GOO was achieved in 87.5% of misdeployment cases (*n* = 7), utilizing either repeat EUS‐GE (*n* = 5) or ES (*n* = 2). After a mean follow‐up of 180 days (standard deviation [SD], 16.7), nonmisdeployment AEs occurred in 4 patients (2.4%), with no procedure‐related mortalities. The reintervention rate was 12.7%, and median stent patency was 767 days (IQR, 389–not reached).

### 3.2 FM and EM Classifications: Interobserver Agreement (Table S1)

Regarding morphological distribution, FM type I was most frequent (55.2%), followed by type III (27.3%), type IV (12.7%), and type II (4.8%). Interobserver agreement for the four FM types yielded a k‐coefficient of 0.874. For EM, type A predominated (47.9%), followed by type B (43.0%) and type C (9.1%). Interobserver agreement of the three EM types showed a *k*‐coefficient of 0.902.

### 3.3 Comparison of Misdeployment and Nonmisdeployment (Table 2; Figure 4)

**FIGURE 4:**
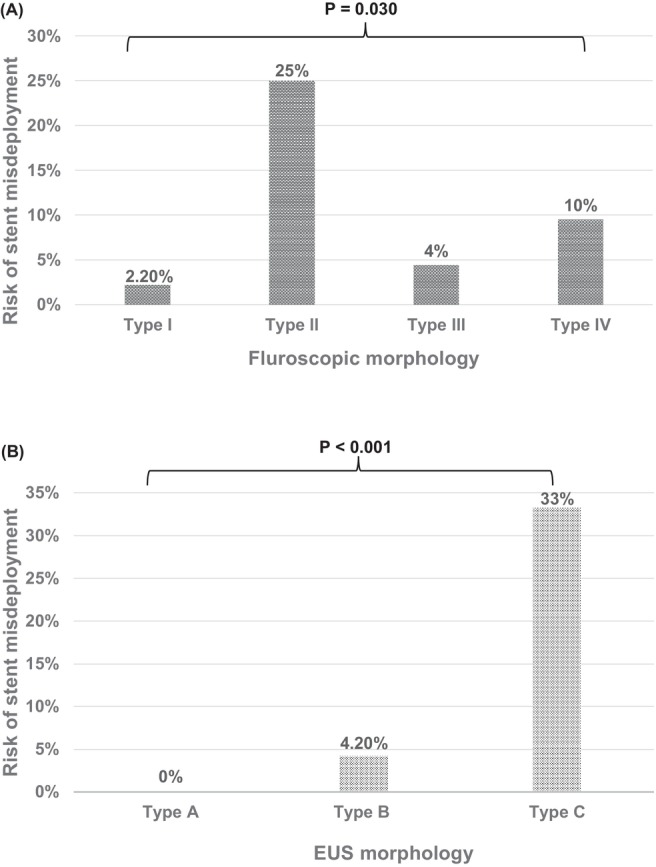
Risk of stent misdeployment based on (A) fluoroscopic morphology and (B) EUS morphology.

Baseline characteristics were comparable between the misdeployment and nonmisdeployment groups. However, the median procedure time was significantly longer in the misdeployment group (87 min; IQR 54–100) than in the nonmisdeployment group (37 min; IQR 29–54; *p* = 0.003). Furthermore, patients with misdeployment had significantly shorter median survival (51 days vs. 121 days; log‐rank *p* = 0.005). Regarding morphologic patterns, FM type II was more prevalent in the misdeployment group than in the nonmisdeployment group (25.0% vs. 3.8%; *p* = 0.030). Similarly, EM type C was significantly more frequent among misdeployment cases (62.5% vs. 6.4%; *p* < 0.001). Notably, no misdeployment events occurred in procedures utilizing the EM type A orientation.

**TABLE 2:** Comparison of outcomes between misdeployment and nonmisdeployment.

	Misdeployment (*n* = 8)	Nonmisdeployment (*n* = 157)	*p*
Male, *n* (%)	4 (50)	78 (49.7)	1
Age, years, mean ± SD	69.1 ± 14.4	66.8 ± 14.1	0.747
BMI, median (IQR)	21 (16.3–25.5)	19.9 (17.7–22.4)	0.617
Albumin, g/dL, mean ± SD	3.1 ± 0.5	3.3 ± 0.6	0.292
ECOG, *n* (%)			
None/mild	3 (37.5)	66 (42)	1
Moderate/severe	5 (62.5)	91 (58)	
Cancer type, *n* (%)			
Pancreatic cancer	6 (75)	103 (65.6)	0.718
Nonpancreatic cancer	2 (25)	54 (34.4)	
Presence of distal metastasis, *n* (%)	4 (50)	93 (59.2)	0.718
Presence of ascites, *n* (%)	2 (25)	71 (45.2)	0.304
Presence of peritoneal seeding, *n* (%)	1 (12.5)	51 (32.5)	0.437
Procedure time, min, median (IQR)	87 (54–100)	37 (29–54)	0.003
Saline injection amount, mL, median (IQR)	1375 (350–2500)	700 (500–1000)	0.359
Fluoroscopic morphology (FM), *n* (%)			
Type I	2 (25)	89 (56.7)	0.030
Type II	2 (25)	6 (3.8)	
Type III	2 (25)	43 (27.4)	
Type IV	2 (25)	19 (12.1)	
EUS morphology (EM), *n* (%)			
Type A	0	79 (50.3)	< 0.001
Type B	3 (37.5)	68 (43.3)	
Type C	5 (62.5)	10 (6.4)	
Follow‐up duration, days, median (IQR)	57 (49–77)	98 (55–212)	0.062
Interval from procedure to reintervention, days, median (IQR)	47 (8–57)	767 (389‐NR)	0.131^a^
Reintervention, *n* (%)	3 (37.5)	18 (11.5)	0.066
Survival length, days, median (IQR)	51 (44–67)	121 (61–239)	0.005^a^

Abbreviations: BMI, body mass index; ECOG, eastern cooperative oncology group; EUS, endoscopic ultrasound; IQR, interquartile range; SD, standard deviation; SM, stent misdeployment.

^a^ Log‐rank test.

### 3.4 Comparison of Outcomes Between the Two Study Periods (Table 3)

Among the initial 85 patients (2020 and 2022), eight misdeployments occurred (9.4%). Following the 2023 implementation of a stepwise target selection strategy, no procedures were aborted due to window identification difficulties, and zero misdeployment events were observed in the subsequent 80 patients (2023–2025; *p* = 0.006). Baseline characteristics were largely comparable, although the 2023–2025 cohort was significantly older (mean 70.2 vs. 63.9 years; *p* = 0.006) with poorer performance status (*p* = 0.002). Despite these higher‐risk profiles, the later group achieved significantly shorter median procedure times (33 vs. 44 min; *p* < 0.001) and required less saline injection (525 vs. 900 mL; *p* < 0.001). Furthermore, median overall survival was significantly longer in the later period (121 vs. 98 days; log‐rank *p* = 0.005).

**TABLE 3:** Comparison of outcomes between study periods.

	2020–2022 (*n* = 85)	2023–2025 (*n* = 80)	*p*
Baseline characteristics			
Male, *n* (%)	46 (54.1)	36 (45)	0.277
Age, years, mean ± SD	63.9 ± 14.6	70.2 ± 12.7	0.006
BMI, median (IQR)	20.1 (17.8–22.7)	19.8 (17.6–22.3)	0.584
Albumin, g/dL, mean ± SD	3.3 ± 0.6	3.2 ± 0.5	0.761
ECOG, *n* (%)			
None/mild	46 (54.1)	23 (28.8)	0.002
Moderate/severe	39 (45.9)	57 (71.2)	
Cancer type, *n* (%)			
Nonpancreatic cancer	26 (30.6)	30 (37.5)	0.412
Pancreatic cancer	59 (69.4)	50 (62.5)	
Presence of distal metastasis, *n* (%)	51 (60)	46 (57.5)	0.754
Presence of ascites, *n* (%)	38 (44.7)	35 (43.8)	1
Presence of peritoneal seeding, *n* (%)	33 (38.8)	19 (23.8)	0.045
Procedure details and outcomes			
Technical success, *n* (%)	82 (96.5)	80 (100)	0.246
Clinical success, *n* (%)	78 (91.8)	79 (98.8)	0.065
Procedure time, min, median (IQR)	44 (35–64)	33 (25–46)	< 0.001
Saline injection amount, mL, median (IQR)	900 (600–1500)	525 (400–875)	< 0.001
Fluoroscopic morphology (FM), *n* (%)			
Type I	46 (54.1)	45 (56.3)	0.226
Type II	7 (8.2)	1 (1.3)	
Type III	22 (25.9)	23 (28.8)	
Type IV	10 (11.8)	11 (13.8)	
EUS morphology (EM), *n* (%)			
Type A	32 (37.6)	47 (58.8)	< 0.001
Type B	38 (44.7)	33 (41.2)	
Type C	15 (17.6)	0	
Follow‐up duration, days, median (IQR)	96 (53–209)	99 (53–210)	0.655
Interval from procedure to reintervention, days, median (IQR)	599 (378‐NR)^a^	767 (476‐NR)^a^	0.292^a^
Reintervention, *n* (%)	14 (16.5)	7 (8.8)	0.164
Adverse event (AE), *n* (%)	12 (14.1)	0	< 0.001
Stent misdeployment	8 (9.4)	0	0.007
Type I misdeployment	4 (2.4)	0	
Type II misdeployment	4 (2.4)	0	
Bleeding	2 (2.4)	0	0.498
Migration	2 (2.4)	0	0.498
ASGE severity grading of AE, *n* (%)			
Mild	9 (75)	0	
Moderate	3 (25)	0	
Survival length, days, median (IQR)	98 (55–209)	121 (61–239)	0.005^a^

Abbreviations: AE, adverse event; BMI, body mass index; ECOG, eastern cooperative oncology group; EUS, endoscopic ultrasound; IQR, interquartile range; NR, not reached; SD, standard deviation.

^a^ Log‐rank test.

### 3.5 Predictors of Misdeployment and Survival (Tables 4 and 5)

Multivariable logistic regression identified EM type C as the sole independent predictor of misdeployment (adjusted OR [aOR], 16.87; 95% CI, 3.17–89.71; *p* = 0.001). Cox regression analysis revealed that misdeployment occurrence was independently associated with diminished survival (adjusted HR [aHR], 2.57; 95% CI, 1.22–5.41; *p* = 0.013). Other independent predictors of worse survival included: pancreatic cancer (aHR, 1.83; 95% CI, 1.22–2.73; *p* = 0.003), malnutrition (aHR, 1.88; 95% CI, 1.29–2.75; *p* = 0.001), poor performance status (aHR, 1.68; 95% CI, 1.16–2.43; *p* = 0.007), presence of ascites (aHR, 1.75; 95% CI, 1.21–2.54; *p* = 0.003), and distant metastasis (aHR, 1.58; 95% CI, 1.11–2.25; *p* = 0.010). Conversely, postprocedural anticancer therapy was associated with improved survival (aHR, 0.54; 95% CI, 0.37–0.79; *p* = 0.002) (Table 5).

**TABLE 4:** Predictors of misdeployment.

	Univariable analysis			Multivariable analysis		
	Odds ratio	95% confidence interval	*p*	Adjusted odds ratio	95% confidence interval	*p*
Sex, male/female	1.01	0.24–4.19	0.986	—	—	—
Age, per 1 year increase	1.01	0.96–1.07	0.651	—	—	—
Moderate/severe vs none/mild ECOG	1.21	0.28–5.24	0.800	—	—	—
Pancreatic vs nonpancreatic cancer	1.57	0.31–8.06	0.587	—	—	—
Ascites, yes/no	0.40	0.08–2.06	0.276	—	—	—
Distal metastasis, yes/no	0.69	0.17–2.85	0.606	—	—	—
Peritoneal seeding, yes/no	0.30	0.04–2.48	0.262	—	—	—
Saline injection amount, > 700 vs ≤ 700 mL	1.73	0.40–7.49	0.463	—	—	—
Fluoroscopic morphology type II vs other types	8.39	1.39–59.56	0.020	7.13	0.82–61.99	0.075
EUS morphology type C vs other types	24.5	5.11–117.5	< 0.001	16.87	3.17–89.71	0.001
Endoscopist experience, ≥ 25 vs < 25 EUS‐GE cases	0.22	0.04–1.14	0.072	0.40	0.06–2.63	0.339

Abbreviations: ECOG, eastern cooperative oncology group; EUS, endoscopic ultrasound; EUS‐GE, endoscopic ultrasound‐guided gastroenterostomy.

**TABLE 5:** Predictors of postprocedure mortality.

	Univariable analysis			Multivariable analysis		
	Hazard ratio	95% confidence interval	*p*	Adjusted hazard ratio	95% confidence interval	*p*
Sex, male/female	0.91	0.65–1.26	0.575	—	—	—
Age, per 1 year increase	1.00	0.99–1.01	0.990	—	—	—
Underweight vs normal/overweight	1.00	0.72–1.39	0.984	—	—	—
Moderate/severe vs none/mild nutrition	1.62	1.15–2.28	0.006	1.88	1.29–2.75	0.001
Moderate/severe vs none/mild ECOG	1.64	1.17–2.29	0.004	1.68	1.16–2.43	0.007
Pancreatic vs nonpancreatic cancer	1.39	0.98–1.97	0.063	1.83	1.22–2.73	0.003
Ascites, yes/no	1.59	1.14–2.20	0.006	1.75	1.21–2.54	0.003
Distal metastasis, yes/no	1.76	1.25–2.46	0.001	1.58	1.11–2.25	0.010
Peritoneal seeding, yes/no	1.16	0.82–1.65	0.398	—	—	—
Anti‐cancer therapy after procedure, yes/no	0.71	0.51–0.99	0.041	0.54	0.37–0.79	0.002
Misdeployment, yes/no	2.74	1.32–5.66	0.007	2.57	1.22–5.41	0.013

Abbreviation: ECOG, eastern cooperative oncology group.

## 4 Discussion

EUS‐GE is an innovative endoscopic technique that provides durable symptom relief for GOO by creating a bypass with a LAMS away from the tumor [1, 2, 3]. Despite its high efficacy [2, 11, 12, 13, 14, 15, 16, 17], misdeployment remains a major barrier to wider adoption. Unlike other interventional EUS procedures targeting fixed structures, EUS‐GE involves a mobile jejunal loop, increasing technical complexity even for experienced endoscopists [4, 5]. Given the sparse evidence regarding risk factors, this prospective study proposes a novel morphological classification—based on fluoroscopic and EUS imaging—to stratify misdeployment risk.

Success in EUS‐GE depends on meticulous patient selection and strict adherence to standardized techniques [4, 18, 19]. In our study, all centers followed the TEGESG protocol, supported by regular quality assurance reviews. Notably, all eight misdeployments in the initial cohort (2020–2022) resulted from distal flange malposition outside the target lumen. Safe deployment requires an adequate intraluminal “runway,” especially within constrained anatomical spaces. Much like the ≥ 12 mm bile duct threshold required for EUS‐guided choledochoduodenostomy [20, 21], EUS‐GE using a 20‐mm LAMS—the most prevalent size in our cohort [22]—necessitates sufficient axial length for distal flange release. While device specifications suggest a required length of approximately 48 mm (Table S2), clinical experience indicates that a practical threshold of > 30–40 mm is typically sufficient for technical success [23].

EM types are defined by the orientation of scanning plane relative to the target bowel lumen. We prioritize parallel orientations (types A and B) over cross‐sectional views (type C). If type C is encountered, it can often be transitioned to a parallel plane by rotating the endoscope. Among parallel views, type A—where the bowel's longitudinal axis is perpendicular to the ultrasound beam—is preferred as it provides an optimal “intraluminal runway”. Our data demonstrated that EM type A was associated with zero misdeployments. Conversely, EM type C provides insufficient spatial orientation and an inadequate runway, resulting in the highest misdeployment rate (33%) and acting as the sole independent predictor of misdeployment (aOR > 15). Consequently, the stepwise strategy implemented in 2023 prioritized EM type A/B while strictly avoiding type C, successfully eliminating misdeployments in the subsequent 80 patients.

Careful selection of the puncture site to minimize the stomach–bowel distance is critical for preventing misdeployment [18]. Although this distance was not systematically quantified in our database, our standardized protocol required endoscopists to confirm a distance of < 10 mm prior to puncture. This ensured compatibility with the 10‐mm saddle length of the LAMS, even in cases with unfavorable anatomy (e.g., FM type II or EM type C).

Building upon the work of Nutahara et al. [24], we propose an FM classification specifically tailored for EUS‐GE in mGOO. Unlike static radiography, our intraprocedural evaluation accounts for the physical alteration of bowel position by the endoscope and nasobiliary tube. These instruments often accentuate “loop” configurations (FM types III and IV) during the procedure. Furthermore, intraluminal distension via contrast and saline can “convert” non‐approaching segments into approachable ones under dynamic conditions. This likely explains the lower prevalence of FM type II in our cohort (4.8%) compared to prior reports. Ultimately, intraprocedural FM reflects the actual anatomical state during intervention, providing a more reliable guide for puncture site selection than pre‐procedural imaging.

Although multivariable analysis identified EM as the sole independent predictor of misdeployment, FM remains essential as a macroscopic “roadmap.” For instance, FM type II or type IV configuration immediately alerts the operator to an increased risk of failure or the necessity for advanced maneuvers, such as deeper catheter advancement to identify bowel segments in closer apposition to the stomach. Therefore, FM and EM are complementary: FM provides the macro‐anatomical context for navigation, while EM ensures micro‐anatomical safety for LAMS deployment.

Our 4.9% misdeployment rate aligns with the 4.6% reported in a recent meta‐analysis of 1846 patients [4]. Notably, misdeployment independently predicted significantly diminished survival (aHR, 2.57; *p* = 0.013), likely because even minor AEs can disproportionately impair prognosis in frail mGOO patients [10, 25, 26]. These findings underscore the clinical imperative for preventive strategies to optimize outcomes. Furthermore, the apparent survival benefit of postprocedural anticancer therapy warrants caution due to potential immortal time bias. To address this, we performed a landmark analysis at 30 days postprocedure. After adjusting for this via a 30‐day landmark analysis, the survival benefit was no longer statistically significant (HR 0.85; 95% CI: 0.59–1.22; *p* = 0.383; Table S4), suggesting that the correlation was driven by selection bias rather than a direct treatment effect.

Despite introducing a novel morphological classification to enhance EUS‐GE safety, several limitations must be acknowledged. First, despite the implementation of a standardized protocol across 13 centers, the risk of misdeployment was likely influenced by accumulated operator experience and technical refinements over time. Consequently, our analysis remains susceptible to selection bias and temporal confounding, particularly when comparing the 2020–2022 and 2023–2025 cohorts. Because the 2023 stepwise strategy mandated avoidance of EM type C, the subsequent absence of misdeployment likely reflects proactive risk avoidance rather than improved management of high‐risk anatomies. Therefore, our classification should be interpreted as a safety‐oriented decision‐making tool to identify high‐risk cases rather than a technique to overcome challenging anatomies. Furthermore, the institutional learning curve over the 5‐year study period cannot be entirely decoupled from the effects of the morphological strategy. In addition, because FM and EM classifications were retrospectively assigned in a subset of earlier cases, information bias cannot be completely excluded. Nevertheless, all classifications were based on archived fluoroscopic and EUS images and demonstrated excellent interobserver agreement, supporting good reproducibility despite the mixed retrospective–prospective design. Second, the limited number of misdeployment events restricted the predictive assessment. Although the Cox regression model may be subject to overfitting, our findings should be interpreted cautiously and serve primarily as a hypothesis‐generating pending further large‐scale validation. Third, the multicenter design and relatively low procedural volume per center mean that inter‐operator variability cannot be entirely excluded. Finally, as this study exclusively utilized the WEST technique, our findings may not be generalizable to other approaches, such as double‐balloon‐assisted methods.

In summary, standardizing procedural protocols through a stepwise target selection strategy—informed by our morphological classification—improves technical performance and reduces adverse events in EUS‐GE. Heightened awareness of high‐risk morphologies, particularly EM Type C, is essential for effective procedural planning and risk stratification. Ultimately, these strategies optimize clinical outcomes and establish a safer framework for the broader adoption of EUS‐GE within the interventional endoscopy community.

## Author Contributions

Y.‐T.K. and H.‐P.W. designed the study. Y.‐T.K. prepared the statistical analysis. Y.‐T.K., C.‐Y.Y., J.‐H.C., S.‐C.L., C.‐L.H., C.‐H.W., M.‐Y.L., C.‐C.C., K.‐C.C., M.‐H.L., H.‐Y.S., J.‐C.L., C.‐K.S., M.‐C.T., and H.‐P.W. recruited patients to the study. Y.‐T.K. drafted the article, which was critically revised by H.‐P.W. All authors commented on drafts and approved the final version. All authors had full access to the data and participated in the decision to submit for publication.

## Funding

The authors have nothing to report.

## Ethics Statement

This study was approved by the ethics committee at each center, and the study was registered at ClinicalTrials.gov (NCT07230665).

## Consent

Informed consents were obtained from all participants.

## Conflicts of Interest

The authors declare no conflicts of interest.

## Supporting information


**Table S1:** Standardized protocol for endoscopic ultrasound‐guided gastroenterostomy (EUS‐GE) by the Taiwan EUS‐GE Study Group (TEGESG).
**Table S2:** Comparison of the required delivery catheter length for distal flange deployment of Hot AXIOS stents by size.
**Table S3:** Number of patients recruited and misdeployment in each center.
**Table S4:** Predictors of 30‐day = postprocedure mortality.
**Figure S1:** Risk of stent misdeployment (SM) based on (A) fluoroscopic morphology (FM) and (B) EUS morphology (EM) in the two study periods.

## Data Availability

The data that support the findings of this study are available from the corresponding author upon reasonable request.
