# Supplementary material for: EUS‐Guided Gastroenterostomy for Malignant Gastric Outlet Obstruction: Morphological Classification to Prevent Stent Misdeployment
Source: Dig Endosc. 2026 Jul 17;38(7):e70235. doi: 10.1111/den.70235 (PMC13378264; doi:10.1111/den.70235)
Supplement: Supplementary file 1 — Table S1: Standardized protocol for endoscopic ultrasound‐guided gastroenterostomy (EUS‐GE) by the Taiwan EUS‐GE Study Group (TEGESG). Table S2: Comparison of the required delivery catheter length for distal flange deployment of Hot AXIOS stents by size. Table S3: Number of patients recruited and misdeployment in each center. Table S4: Predictors of 30‐day = postprocedure mortality. Figure S1: Risk of stent misdeployment (SM) based on (A) fluoroscopic morphology (FM) and (B) EUS morphology (EM) in the two study periods. [file DEN-38-0-s001.docx]

**Supplementary material**

EUS-guided gastroenterostomy for malignant gastric outlet obstruction: morphological classification to prevent stent misdeployment

Yu-Ting Kuo, Chi-Ying Yang, Jiann-Hwa Chen, Szu-Chia Liao, Cheng-Lin Hsieh, Chia-Hsien Wu, Meng-Ying Lin, Chen‐Shuan Chung, Kuan-Chih Chen, Mu-Hsien Lee, Hsiang-Yao Shih, Jung-Chun Lin, Cheuk-Kay Sun, Ming-Chang Tsai, Hsiu-Po Wang, on behalf of the Taiwan EUS-GE Study Group (TEGESG)

**Table 1.** Standardized protocol for endoscopic ultrasound-guided gastroenterostomy (EUS-GE) by the Taiwan EUS-GE Study Group (TEGESG)

| **Pre-procedure care** |
| --- |
| - Labs: complete blood count (CBC), prothrombin time–international normalized ratio (PT-INR), routine chemistry |
| - Cross-sectional imaging within 30 days prior to procedure |
| - Nasogastric tube decompression if large amount of retained intra-gastric food contents on cross-sectional imaging |
| - Ensure NPO status 12 hours prior to procedure |
| - Reverse anticoagulation prior to procedure |
| - Single dose of intravenous prophylactic antibiotics |
| - Record baseline information, BMI, diagnosis, location of GOO, tumor status, GOOS score, and ECOG score |
| **Equipment and room setup during the procedure** |
| - General anesthesia with endotracheal intubation |
| - Endoscopic ultrasound and linear echoendoscope |
| - Duodenoscope or forward-viewing therapeutic endoscope |
| - Fluoroscopy |
| - Electrosurgical generator with pure cut (Such as ERBE system: auto cut, 120 watts, effect 5) |
| - Carbon dioxide insufflation |
| - 20-mm extraction balloon catheter or standard ERCP cannula |
| - 0.025- or 0.035-inch guidewire |
| - 7Fr nasobiliary tube |
| - Saline mixture with contrast medium and indigo carmine |
| - Standard infusion pump |
| - Glucagon 1mg |
| - Hot AXIOS, 20 mm diameter, 10 mm length |
| **Record details of the procedure** |
| - Total procedure time |
| - Technical success |
| - Location of LAMS placement |
| - Total amount of injected saline mixture |
| - Fluoroscopic morphological classification of the gastrointestinal tract around the ligament of Treitz |
| - EUS-based morphological classification of the targeted bowel loop |
| - Adverse event |
| **Post-procedure care** |
| - Overnight hospitalization post-procedural monitoring |
| - Avoid anticoagulation for 48 hours |
| - POD 1: Oral refeeding with a clear liquid diet began 4 to 6 hours post-procedure |
| - POD 2: Soft diet |
| - Since POD 3: low residue or normal diet, if tolerate |
| - Follow up abdominal X-ray on POD 1, POD 2, and POD 3 |
| - First outpatient clinic follow-up within 4 weeks post-discharge |
| - Outpatient clinic follow-up every three months until death |
| - Record BMI, GOOS score, and ECOG score during each clinic visit |

BMI, body mass index; ECOG, eastern cooperative oncology group; EUS-GE, endoscopic ultrasound-guided gastroenterostomy; ERCP, endoscopic retrograde cholangiopancreatography; GOO, gastric outlet obstruction; GOOS, gastric outlet obstruction score; IQR, interquartile range; LAMS, lumen-apposing metal stent; NPO, nothing by mouth; POD, postoperative day

**Table 2.** Comparison of the required delivery catheter length for distal flange deployment of Hot AXIOS stents by size

| **Size of Hot AXIOS**  (Stent diameter [mm] x Saddle length [mm]) | **Length of the Delivery Catheter**  **Required for Distal Flange Deployment**  (cm) | |
| --- | --- | --- |
| **6 x 8** | 2.6 | 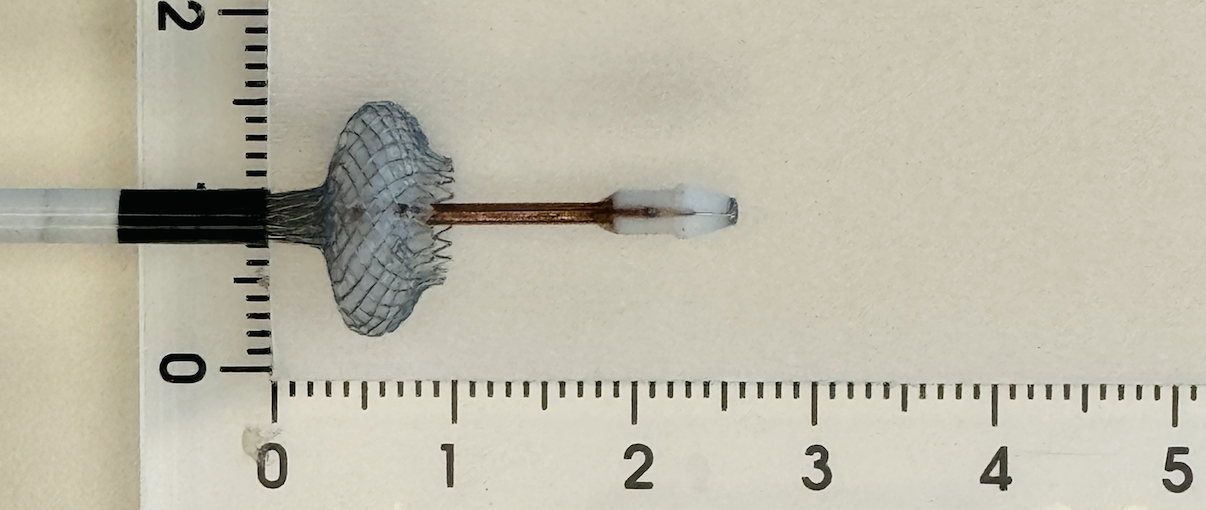 |
| **8 x 8** | 3.4 | 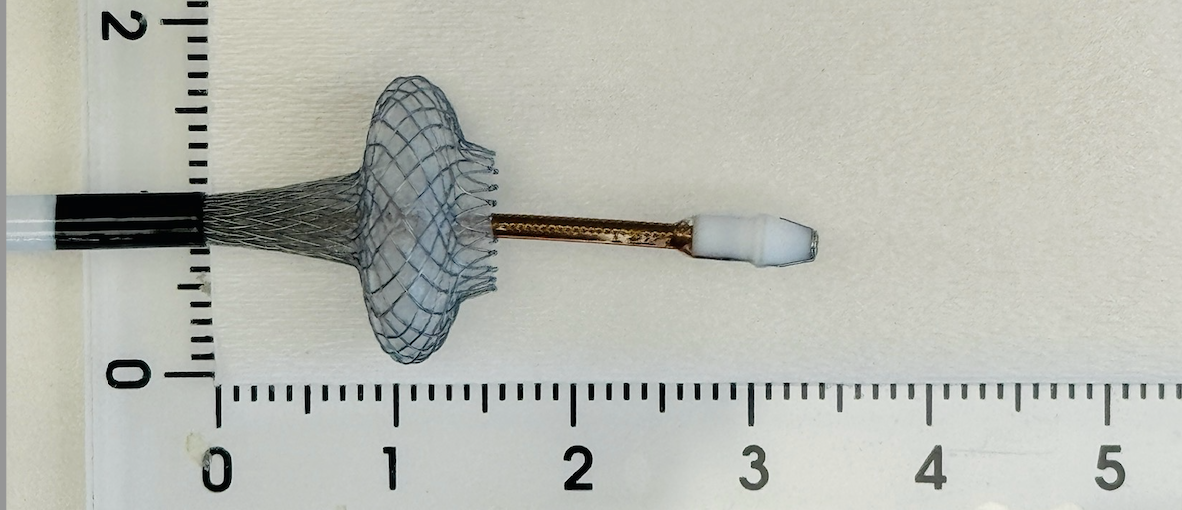 |
| **10 x 10** | 4.4 | 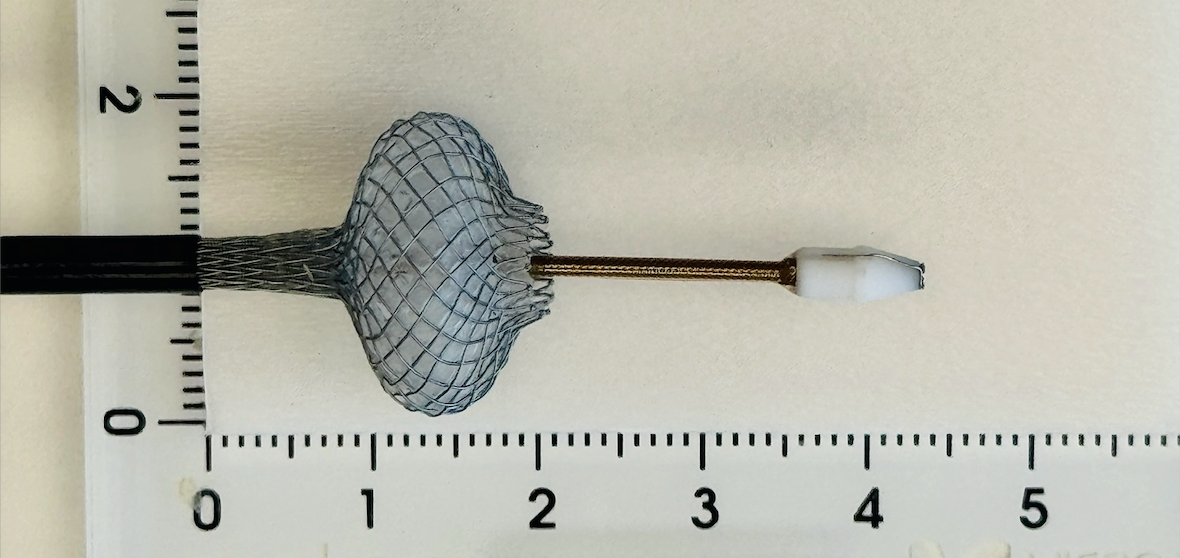 |
| **15 x 10** | 4.4 | 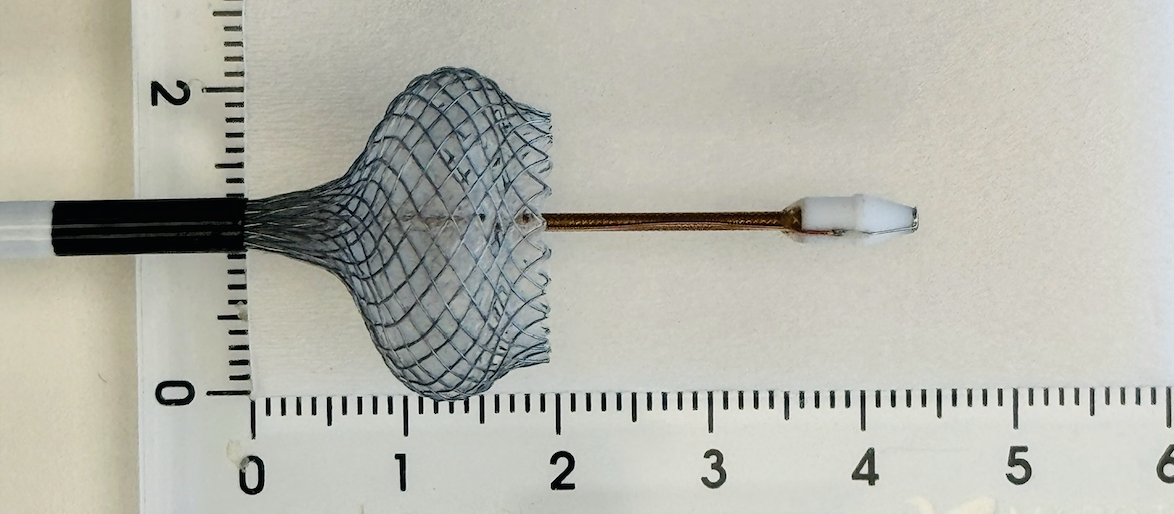 |
| **15 x 15** | 4.8 | 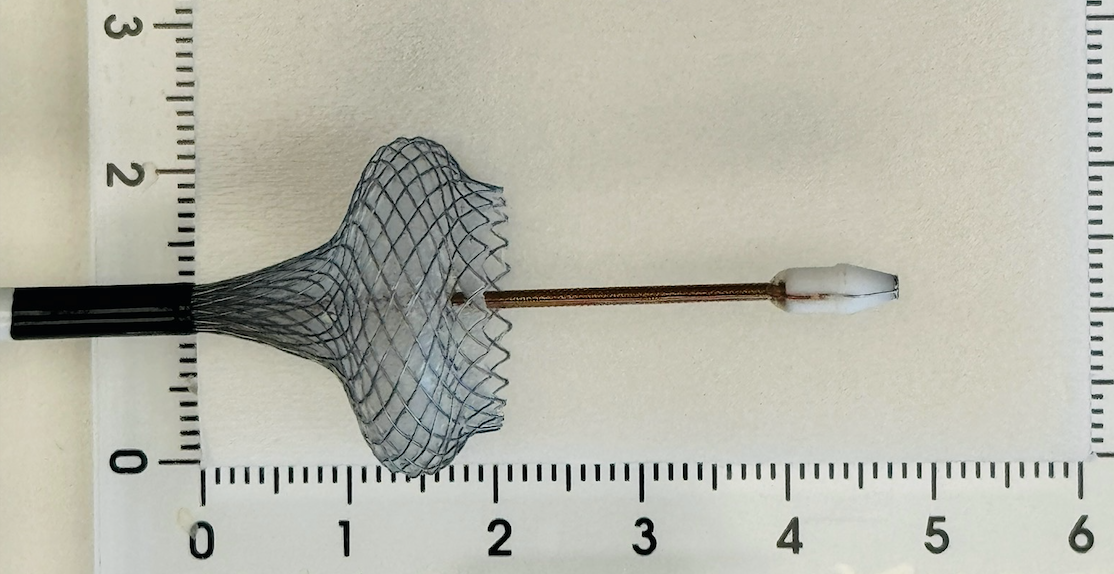 |
| **20 x 10** | 4.8 | 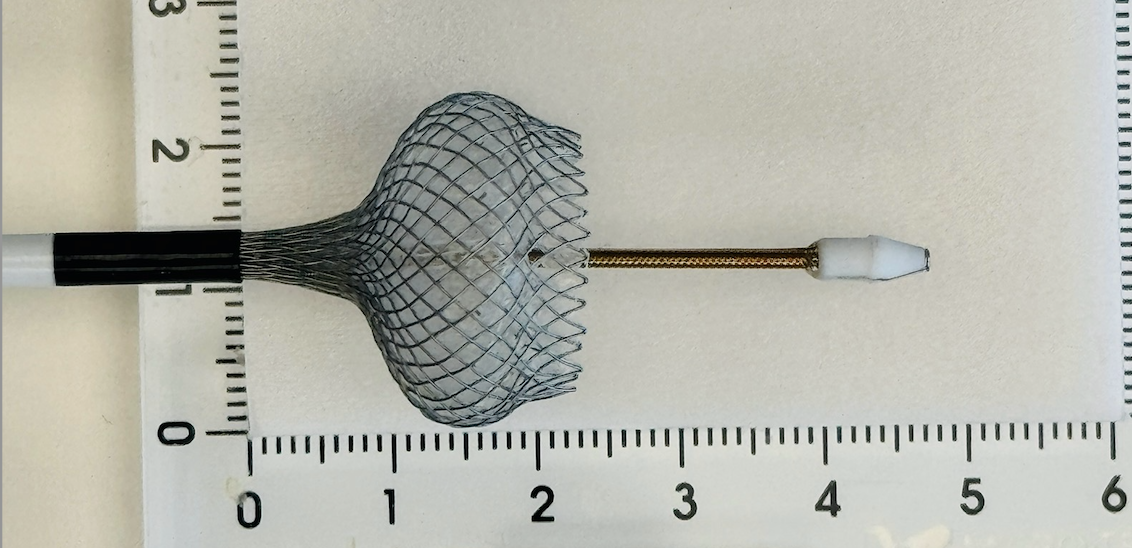 |

**Table 3. Number of patients recruited and misdeployment in each center.**

|  | **EUS-GE**  **(N=165)** | **Misdeployment**  **(N=8)** |  |
| --- | --- | --- | --- |
| Hospital  National Taiwan University Hospital  China Medical University Hospital  Taipei Tzu Chi Hospital  Taichung Veterans General Hospital  National Taiwan University Hospital Hsin-Chu Branch  National Cheng Kung University Hospital  Far Eastern Memorial Hospital  Taitung Mackay Memorial Hospital  Chang Gung Memorial Hospital  Shin Kong Wu Ho-Su Memorial Hospital  Kaohsiung Medical University Hospital  Chung Shan Medical University Hospital  Tri-Service General Hospital | 104  13  10  9  5  5  5  4  4  2  2  1  1 | 3  2  1  0  0  1  0  0  0  0  0  0  1 | 22^rd^, 31^st^, and 48^th^ cases  6^th^ and 8^th^ cases  2^nd^ case  4^th^ case  1^st^ case |

**Table 4.** Predictors of 30-day= post-procedure mortality.

|  | **Univariable analysis** | | | **Multivariable analysis** | | |
| --- | --- | --- | --- | --- | --- | --- |
|  | **HR** | **95% CI** | ***P* value** | **aHR** | **95% CI** | ***P* value** |
| **Sex, male/female** | 0.93 | 0.66 – 1.31 | 0.683 | - | - | - |
| **Age, per 1 year increase** | 1.00 | 0.99 – 1.01 | 0.875 | - | - | - |
| **Underweight vs normal/overweight** | 1.04 | 0.74 – 1.48 | 0.791 | - | - | - |
| **Moderate/severe vs none/mild nutrition** | 1.62 | 1.13 – 2.32 | 0.009 | 1.61 | 1.09 – 2.36 | 0.016 |
| **Moderate/severe vs none/mild ECOG** | 1.57 | 1.11 – 2.23 | 0.011 | 1.86 | 1.28 – 2.70 | 0.001 |
| **Pancreatic vs nonpancreatic cancer** | 1.30 | 0.91 – 1.87 | 0.148 | 1.43 | 0.96 – 2.14 | 0.081 |
| **Ascites, yes/no** | 1.57 | 1.11 – 2.22 | 0.010 | 1.67 | 1.13 – 2.46 | 0.010 |
| **Distal metastasis, yes/no** | 1.64 | 1.16 – 2.33 | 0.006 | 1.52 | 1.06 – 2.19 | 0.023 |
| **Peritoneal seeding, yes/no** | 1.12 | 0.77 – 1.61 | 0.562 | - | - | - |
| **Anti-cancer therapy after procedure, yes/no** | 0.85 | 0.59 – 1.22 | 0.383 | - | - | - |
| **Misdeployment, yes/no** | 3.02 | 1.39 – 6.56 | 0.005 | 3.30 | 1.49 – 7.35 | 0.003 |

ECOG, eastern cooperative oncology group; HR, hazard ratio; 95% CI, 95% Confidence interval; aHR, adjusted hazard ratio

**Figure 1.** Risk of stent misdeployment (SM) based on (**A**) fluoroscopic morphology (FM) and (**B**) EUS morphology (EM) in the two study periods.

**(A)**


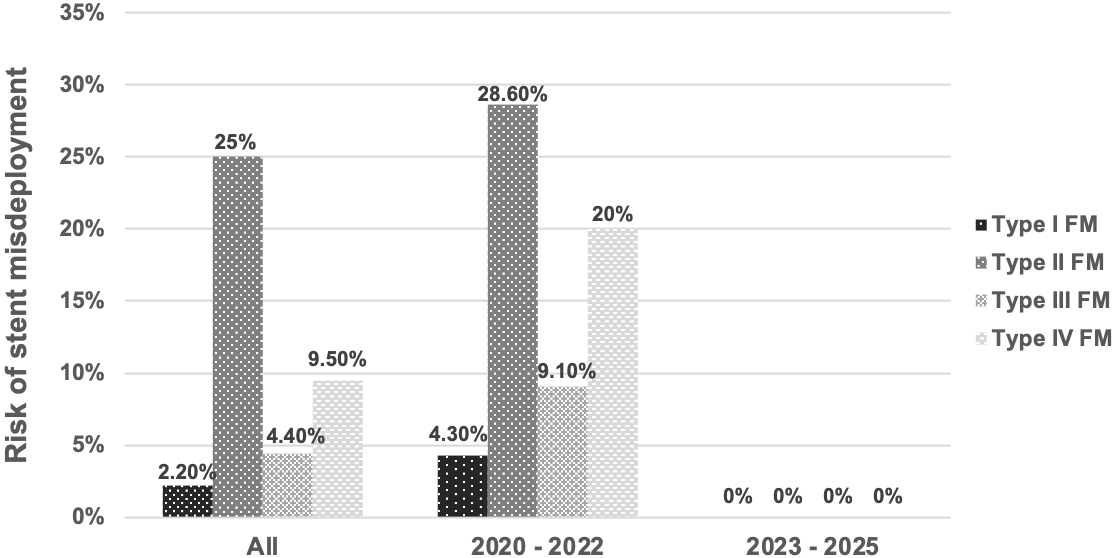


**P = 0.073**

**P = 0.030**


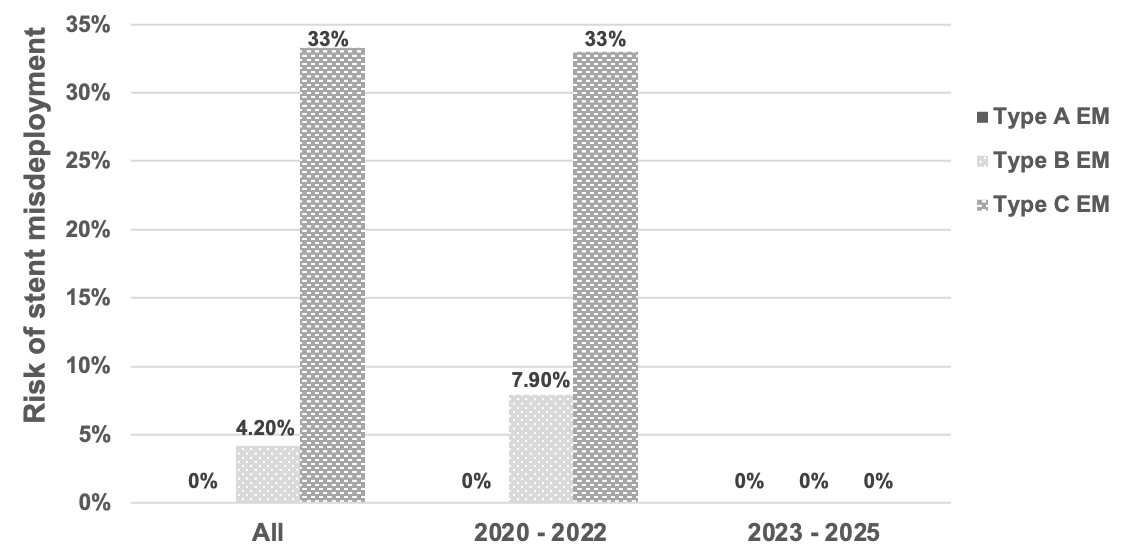


**P < 0.001**

**P = 0.001**

**(B)**
